# Identification and Characterization of Planktonic Biofilm-Like Aggregates in Infected Synovial Fluids From Joint Infections

**DOI:** 10.3389/fmicb.2020.01368

**Published:** 2020-06-30

**Authors:** Alessandro Bidossi, Marta Bottagisio, Paolo Savadori, Elena De Vecchi

**Affiliations:** ^1^Laboratory of Clinical Chemistry and Microbiology, IRCCS Istituto Ortopedico Galeazzi, Milan, Italy; ^2^Department of Endodontics, IRCCS Istituto Ortopedico Galeazzi, Milan, Italy

**Keywords:** joint infections, biofilm, *Staphylococcus*, synovial fluid, aggregates

## Abstract

Recent *in vitro* studies reported the exceptional ability of some bacterial species to form biofilm-like aggregates in human and animal synovial fluids (SF), but evidences from infected clinical samples are still lacking. In this study, we investigated whether this bacterial phenotype was present in infected SFs collected from joint infections and if it was maintained in *in vitro* settings. SFs sent for culture to the Laboratory of Microbiology of our institute were directly analyzed by means of confocal laser scanning microscopy (CLSM), and the infective agents were isolated for further *in vitro* tests. Moreover, sterile SF was collected from patients who did not receive previous antibiotic therapy to investigate the formation of bacterial aggregates, together with biofilm and matrix production on a titanium surface. Finally, antibiotic susceptibility studies were performed by using bovine SF. Four *Staphylococcus aureus*, one *Staphylococcus lugdunensis*, and one *Prevotella bivia* strain were identified in the infected SFs. The CLSM analysis showed that all staphylococci were present as a mixture of single cells and bacterial clumps surrounded by an exopolymeric substance, which comprised SF-derived fibrin, while all *P. bivia* cells appeared separated. Despite that, differences in the ability to aggregate between *S. aureus* and *S. lugdunensis* were observed in clinical SFs. These different phenotypes were further confirmed by *in vitro* growth, even though the application of such *ex vivo* approach lead all staphylococci to form exceptionally large microbial aggregates, which are several folds bigger than those observed in clinical samples. Planktonic aggregates challenged for antibiotic susceptibility revealed a sharp increase of recalcitrance to the treatments. Although this is still at a preliminary stage, the present work confirmed the ability of staphylococci to form free-floating biofilm-like aggregates in infected SF from patients with joint infections. Furthermore, the obtained results pointed out that future *in vitro* research on joint infections will benefit from the use of human- or animal-derived SF. Even though this approach should be carefully validated in further studies comprising a larger microbial population, these findings pose new challenges in the treatment of infected native and prosthetic joints and for the approach to new investigations.

## Introduction

The analysis of synovial fluid (SF) has long been recommended as a routine procedure in the diagnosis of joint infections. Septic native joints are quite uncommon, with approximately two cases per 100,000 people per year (Ross, 2017), but this event may lead to joint damage and consequent disability. The presence of a prosthetic device drastically increases both the morbidity and the mortality of septic arthritis up to more than 3%, which accounts for almost a third of all the causes for implant revision (Beam and Osmon, 2018). Also, the etiology of native and prosthetic joint infections differs. While in the first case *Staphylococcus aureus* is responsible for more than 50% of the cases, prosthetic joint infections (PJIs) see a very high incidence of low-virulence microorganisms, such as coagulase-negative staphylococci (CoNS) and anaerobes such as *Cutibacterium acnes* (Drago et al., 2017). Indeed the pathogenicity of such opportunistic species mainly relies on their ability to adhere to abiotic surfaces and to form mature biofilm by producing or incorporating polymeric substances (Boyle et al., 2018). As a consequence of this protected mode of growth, the sessile microbes become resilient to antibiotics and the immune system. Furthermore, contrarily to isolated planktonic bacteria, they often fail to grow in standard culture conditions used in diagnostics (Costerton et al., 2011; Dastgheyb et al., 2015a).

Although most of the attention has been paid to biofilms attached to implanted devices, recent direct microscopic analysis of clinical samples from diverse chronic infections revealed that bacterial cells physically aggregated in free-floating clusters (Bjarnsholt et al., 2009; Homøe et al., 2009; Burmølle et al., 2010; Bay et al., 2018). Although such cellular aggregates do not need a surface to form, they exhibit many characteristics of sessile biofilms. Indeed they displayed similar growth rates, resistance to phagocytosis, polymeric matrix components, and a reduced antibiotic susceptibility, which can be restored upon disruption (Alhede et al., 2011).

Evidences of bacterial aggregates in infected joint aspirates from clinical settings are scarce. To our knowledge, only a case report published in 2008 described large *S. aureus* aggregates in the SF of an infected prosthetic elbow, but their presence was briefly discussed as “clumps of bacteria that had shed naturally from the biofilm” (Stoodley et al., 2008). Since then, a few other *in vitro* studies investigated the bacterial behavior and the phenotypes in human and animal SF, focusing their attention mainly on *S. aureus* and CoNS and reporting the exceptional ability of these species to form macroscopic clumps (Dastgheyb et al., 2015a,b,c; Perez and Patel, 2015; Ibberson et al., 2016; Gilbertie et al., 2019). These studies highlighted a multifactorial process of aggregation that involves avid binding of fibrin and fibronectin by means of surface adhesins (Dastgheyb et al., 2015a), incorporation of hyaluronic acid (Ibberson et al., 2016), and uncontrolled accumulation of biomass due to the negative regulation of phenole-soluble modulin, which is responsible for disrupting the interactions between bacterial-derived matrix and cells (Dastgheyb et al., 2015c). As a consequence, the staphylococcal aggregates display a superior antimicrobial tolerance that far exceeds the concentrations that would normally kill or inhibit their planktonic counterparts (Gilbertie et al., 2019). This phenomenon is partially restored by the dispersal activity of enzymes which are active on the matrix components (Dastgheyb et al., 2015a; Ibberson et al., 2016; Gilbertie et al., 2019).

The aim of the present work was to identify the presence of bacterial clumps in SFs collected from the infected joints of patients referred to our institute and to evaluate the extent of cellular aggregation, the presence of matrix components, and the relative impact on antimicrobial treatment. For this purpose, the SFs from the diagnostic laboratories of our institute were stained and directly analyzed by means of confocal laser scanning microscopy (CLSM), and the infective agents were isolated and employed for further *in vitro* tests.

## Materials and Methods

### Synovial Fluid Collection

Human SFs were collected from patients referred to the Orthopedic Institute Galeazzi, with suspected joint infections, in accordance with a human subject protocol approved by the San Raffaele Hospital Ethical Committee (No. 146/int/2018). A written consent was signed by each patient before the SF aspiration. The SFs, either collected from outpatients or from the operating room, were firstly brought to the Laboratory of Microbiology of the institute to undergo routine diagnostic procedures. After routine processing, an aliquot was directly observed by CLSM. Thereafter, the infective agents were isolated for the subsequent *in vitro* tests. The non-infected SFs from patients who did not receive any previous antibiotic therapy were employed to investigate the *in vitro* formation of planktonic aggregates, biofilm formation on titanium surface, and matrix production. To do that, sterile SFs were decellularized by centrifugation at 3,000 rpm for 10 min and stored at −20°C. All the sterile SFs were pooled before the *in vitro* experiments.

### Bacterial Strains

The identification of clinical isolates was carried out on a Vitek2 Compact (BioMerieux) and later confirmed by sequencing of 80 bp of the variable regions V1 and V3 of the 16S rRNA gene by Pyrosequencing (PSQ96RA, Diatech). All strains were stored at −80°C in brain heart infusion (BHI, Merck) broth enriched with 10% glycerol (Sigma Aldrich) until testing. Before the *in vitro* experiments, the bacteria were thawed and reconstituted in tryptic soy agar (Biomérieux) for 24 h at 37°C. *Prevotella bivia* was grown on Schaedler agar (Oxoid) plus 3% defibrinated sheep blood (Thermofisher) and incubated in anaerobiosis for at least 48 h.

### Confocal Microscopy on Clinical SF

All SF samples were immediately analyzed when possible or fixed with 2% paraformaldehyde solution. A 50-μl aliquot was stained for 15 min in the dark by adding the same volume of a solution containing 0.5 μM of SYTO 9 Green Fluorescent Nucleic Acid Stain (Thermo Fisher Diagnostics SpA). After incubation, the samples were spread over the surface of a glass slide, air-dried, and then observed with an upright TCS SP8 (Leica Microsystems CMS GmbH) CLSM. When needed, the samples were diluted with sterile saline. Only the samples containing bacterial aggregates were further stained to identify self-produced or incorporated extracellular matrix components. In particular, BOBO-3 (Invitrogen), a cell-impermeable DNA stain, was employed at a final concentration of 100 nM to visualize extracellular DNA (eDNA). Wheat-germ agglutinin (WGA) Alexa Fluor 647 conjugate (Invitrogen; final concentration, 5 μg/ml), a lectin that binds to N-acetyl-d-glucosamine and N-acetylneuraminic acid residues, was used to stain staphylococcal poly-N-acetylglucosamine (PNAG) (Frank and Patel, 2007) or SF-derived hyaluronic acid incorporated in bacterial aggregates (Ibberson et al., 2016). FilmTracer SYPRO Ruby Biofilm Matrix Stain (Invitrogen) was added at 1:1 to the sample, allowed to stain for 30 min, and then gently washed three times with sterile saline to examine matrix proteins. Finally, anti-fibrin alpha chain antibody conjugated to Cy5 (Abcore Inc.) was used (50 μg/ml) to visualize the presence of human fibrin in the clumps. The images were then acquired using a × 20 dry objective (HC PL FLUOTAR 20×/0.50 DRY) or a × 100 immersion oil objective (HC PL APO 100X/1.40 OIL CS2) and processed with Las X software (Leica Microsystems CMS GmbH).

### Biofilm-Like Aggregates and Biofilm Formation on Titanium Discs

The clinical strains were tested *in vitro* for their ability to form aggregates and to attach to the titanium surface. Next, 5-mm-diameter titanium alloy disks (Ti6Al4V, Geass) were placed in a flat-bottom 96-well microplate (VWR) and covered with 180 μl of either sterile SF or BHI (in the case of staphylococci) or thioglycollate broth (for *P. bivia*). The clinical strains reconstituted from an overnight culture were resuspended in sterile saline solution to a turbidity of 0.5 McFarland, from which 20 μl was inoculated in each well to reach a final bacterial concentration of ∼10^7^ CFU/ml. The staphylococci were then incubated overnight at 37°C on a shaker at 200 rpm, whereas *P. bivia* was incubated in anaerobiosis for up to 14 days. Thereafter, the supernatant containing aggregated planktonic cells was gently removed with a Pasteur pipette and divided into 50-μl aliquots for CLSM staining (i.e., BOBO-3, WGA, SYPRO Ruby, and anti-fibrin alpha chain antibody conjugated to Cy5), as described in the previous section. The surface of titanium disks was also attentively analyzed by means of CLSM. Briefly, the titanium coupons were washed three times with sterile saline, and 30 μl of staining solution, containing the abovementioned dyes, was applied all over the surface for 30 min in the dark, then washed again, and allowed to dry until the CLSM analysis. The images were acquired as described in the previous paragraph. Each strain was tested in triplicate for each condition, and at least three images were acquired for each replicate. All the acquisitions were performed on the same day using the same stain solutions and applying the same software settings for each sample. Each matrix component was quantified by means of Fiji software (Fiji, ImageJ) and reported as the relative increase in volume with respect to cellular biomass (SYTO 9), indicated as 1 in the scale bar.

### Dispersal Treatment of Sessile Biofilms on Titanium Surface and Planktonic Aggregates

The efficacy of chemical or enzymatic treatments to disperse cells within the biofilm matrix was evaluated. To perform this *in vitro* analysis, bovine synovial fluid (BSF) was purchased from Lampire Biological Laboratories since the ability of human pathogens to form aggregates in animal-derived SF was already observed (Perez and Patel, 2015; Gilbertie et al., 2019). Despite the evidence reported in the literature, the ability of the tested clinical isolates to form planktonic biofilm-like aggregates in this BSF was evaluated prior to proceeding with further *in vitro* experiments.

The staphylococcal clinical isolates were grown on titanium disks with either BSF or BHI as described in the paragraph above. After 24 h of incubation, the titanium disks were removed from the wells, gently rinsed three times with sterile saline, and placed in a new 96-well microplate. Afterward, 200 μl of sterile saline containing either proteinase K (100 μg/ml, Sigma Aldrich) or DNase I (200 μg/ml, Thermo Scientific) or sodium metaperiodate (NaIO_4_, 10 nM, Sigma Aldrich) or plasmin (150 μg/ml, Sigma Aldrich) was added to each well to test the efficacy of each dispersal treatment. Similarly, planktonic aggregates cultured in BSF for 24 h under agitation were treated by adding the same dispersal agents to reach the concentrations indicated above. The microplates containing both treated titanium disks and planktonic aggregates were then placed at 37°C on a shaker at 200 rpm for 1 h, and the CFU increase was counted by plating appropriate dilutions on agar plates. Four replicates for each treatment were performed for each of the tested strain.

### Antibiotic Treatment Exposure

Susceptibility to antibiotics commonly employed in orthopedics was assessed by determining the minimum inhibitory concentration (MIC) and the minimum bactericidal concentration (MBC) by broth microdilution method. In particular, the antibiotic susceptibility of the tested clinical isolates was assessed either in the presence of culture broths [Mueller Hinton for staphylococci (Conda) and thioglycollate (Oxoid) for *P. bivia*] or BSF. In addition, antibiotic susceptibility was investigated on pre-formed bacterial aggregates cultured in BSF, as previously described. To do that, preliminary tests were conducted to standardize the experimental conditions in order to obtain bacterial aggregates with the characteristics previously observed in the clinical scenario. The strains took ∼6 h to form aggregates and clumps with a size similar to that observed in the clinical specimens. At the end of the 6 h incubation period, the cellular load of each strain was quantified in order to standardize the initial inoculum and make the three modalities of antibiotic susceptibility comparable. With this aim, a bacterial suspension of ∼10^5^ CFU/ml was inoculated in BSF and incubated at 37°C on a shaker at 200 rpm. At the end of the 6-h incubation period, two approaches were adopted for the quantification of the final bacterial concentration. First of all, the aliquots of samples were sonicated in a sonication bath (Ultrasonic Bath, VWR) for 5 min at 45 kHz and incubated in agitation for 30 min at 37°C with proteinase K (100 μg/ml). Afterward, the CFUs were counted by plating appropriate dilutions on agar plates. Furthermore, to assess the efficacy of the dispersal treatment, Gram staining of treated and untreated samples was performed. Simultaneously, a second quantification was performed by means of the resazurin assay, as described by Ravi et al. (2019). Briefly, a standard plate containing 1-fold difference of a known cellular concentration between rows and 10-fold difference between columns was prepared from an overnight culture in a 96-well microplate for the fluorescence-based assays (Invitrogen). Resazurin (Alamar Blue, Invitrogen) was added to each well to reach a concentration of 40 μg/ml, and the mixture was incubated in the dark for 2 h. Fluorescence was read at a wavelength of 600 nm with a spectrophotometer (VICTOR X3, Perkin Elmer). Similarly, three aliquots from a 6-h bacterial culture in BSF of each strain were transferred in a microplate. After incubation with resazurin, fluorescence was read and compared to the values obtained by standard plate reading. These results allowed the assessment of MIC and MBC of both planktonic cells and bacterial aggregates following the Clinical and Laboratory Standards Institute guidelines (Clinical and Laboratory Standards Institute guidelines [CLSI], 2015). Briefly, a 0.5 McFarland suspension (about 1.5 × 10^8^ CFU/ml) was prepared in sterile saline, further diluted to reach the appropriate inoculum load, and inoculated in a 96-well microtiter plate containing serial 2-fold dilutions of oxacillin (SigmaAldrich), levofloxacin (Alfa Aesar), and rifampin (Acros Organics) for staphylococci and imipenem (Supelco), ampicillin (Fisher Bioreagents), and metronidazole (Supelco) for *P. bivia*. Vancomycin (PanReac AppliChem ITW Reagents) was tested on all the strains. To investigate the antimicrobial susceptibility of the preformed aggregates, 50 μl of bacterial suspension of ∼10^5^ CFU/ml in BSF was added to the wells of a microplate and incubated at 37°C on a shaker at 200 rpm for 6 h. In another plate, a twofold serial dilution of the aforementioned antibiotics was prepared in BSF. At the end of the incubation, 50 μl from each well containing the serially diluted antibiotic was transferred to the wells containing the preformed bacterial aggregates and incubated. The MIC values, corresponding to the lowest antimicrobial concentration able to inhibit any visible bacterial growth, were read after 24 h of incubation at 37°C. The plates containing the aggregates in BSF were centrifuged to let the bacterial clumps deposit on the bottom of the wells so as to clearly establish the growth. MBC was determined by plating 10 μl from each well, showing no turbidity onto the agar plates. Due to the possible presence of residual clumps of cells, before plating, the microplates were sonicated for 5 min at 45 kHz and incubated in agitation for 30 min at 37°C with proteinase K (100 μg/ml). After incubation, MBC was read as the lowest concentration able to kill 99.9% of the initial inoculum.

### Statistical Analysis

Statistical analyses were performed by means of Graph Pad Prism 5 software (Graph Pad Software, Inc., La Jolla, CA, United States). Shapiro–Wilk test was used to assess the distribution of data. Quantification of matrix composition and evaluation of dispersal treatments were analyzed using one-way non-parametric Kruskal–Wallis test and *post hoc* Dunnett’s test. For comparison of volume/surface ratio on titanium in the presence of BHI medium or SF, a pairwise comparison between the means of the two groups was performed by means of two-tailed, unpaired Student *t*-test for each strain. *P*-values < 0.05, < 0.01, and < 0.001 were considered as significant.

## Results

### Biofilm-Like Aggregates Are Formed by Staphylococci but Not *P. bivia* in Infected SF

**FIGURE 1:**
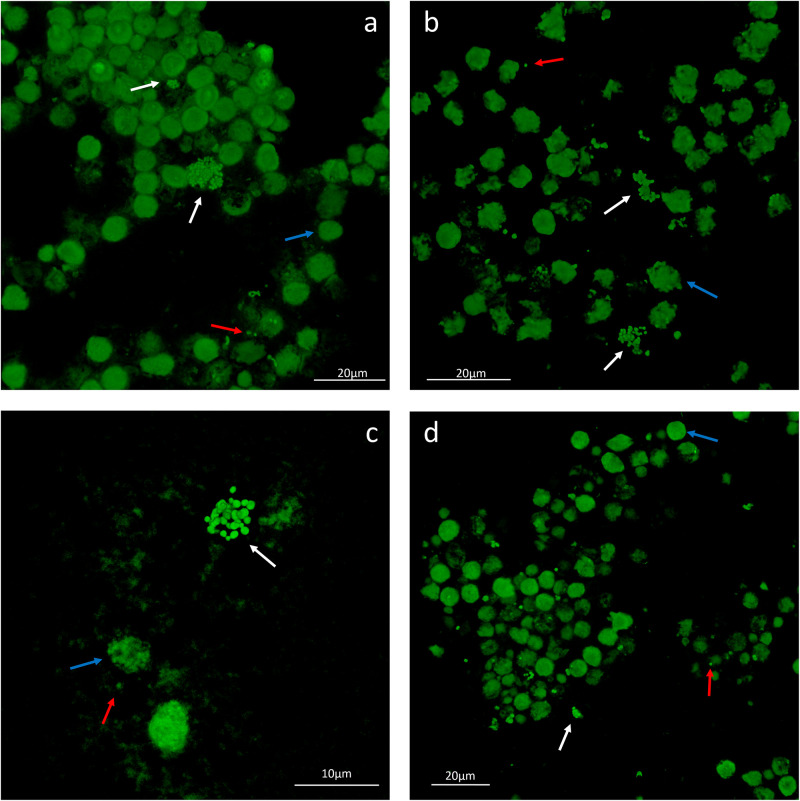
Representative confocal laser scanning microscopy images of infected synovial fluids stained with SYTO 9 nucleic acid stain. **(a)** *Staphylococcus aureus* 1, **(b)** *S. aureus* 2, **(c)** *S. aureus* 3, and **(d)** *Staphylococcus lugdunensis*. The white arrows indicate the biofilm-like aggregates of cells, the red arrow indicates the single bacterial cells, and the blue arrows indicate the immune cells nuclei.

**FIGURE 2:**
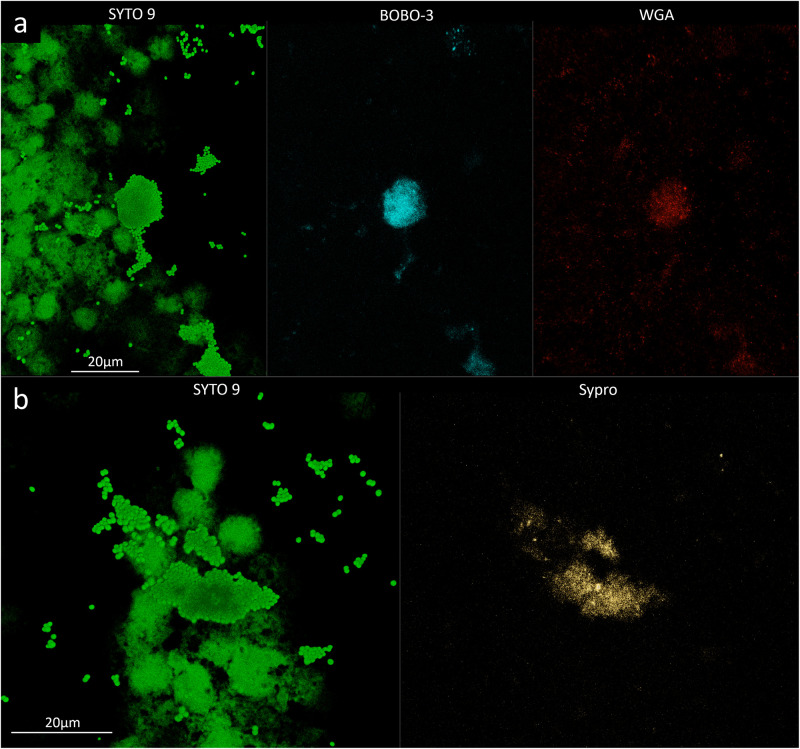
Confocal laser scanning microscopy analysis of synovial fluid infected by *Staphylococcus aureus* 4. **(a)** Sample stained with SYTO 9 for cellular biomass (green, left panel), BOBO-3 for eDNA (light blue, central panel), and WGA stain for GlcNAc containing polysaccharides (red, right panel). **(b)** Sample stained with SYTO 9 for cellular biomass (green, left panel); SYPRO-Ruby staining shows the proteins contained in the biofilm matrix (yellow, right panel).

**FIGURE 3:**
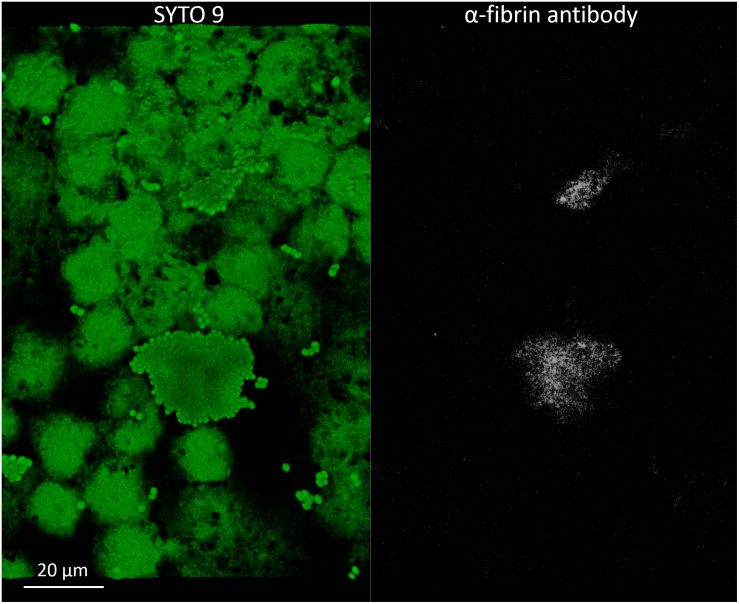
α-Fibrin antibody directly detects fibrin incorporated into the matrix of *Staphylococcus aureus* 4 biofilm-like aggregates in infected synovial fluid clinical sample. Left panel, SYTO 9 for cellular biomass (green); right panel, α-fibrin antibody (gray).

**FIGURE 4:**
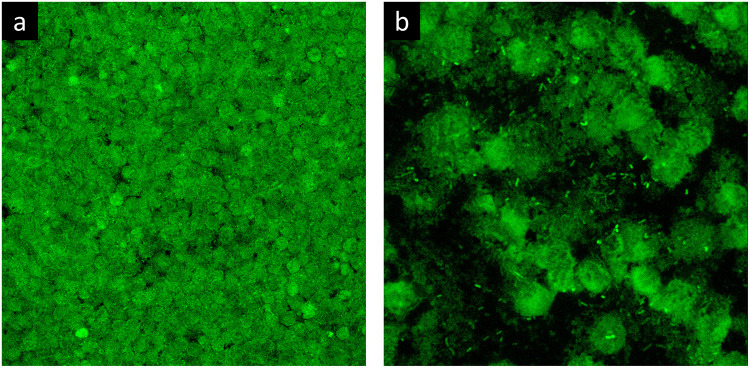
Confocal laser scanning microscopy analysis of synovial fluid infected by *Prevotella bivia* stained with SYTO 9. **(a)** Undiluted SF and **(b)** SF diluted 1:10.

To evaluate the phenotype of bacterial cells in SF, joint aspirates from patients with suspected infection were directly investigated by means of CLSM. Of the 20 synovial fluids with suspected infection, six showed a marked presence of bacterial cells along with massive infiltration of immune cells, which are clearly visible due to the nucleic acid stain SYTO 9 (Figure 1). All specimens exhibited a monomicrobial infection: four were caused by *S. aureus* (one was methicillin resistant and three were methicillin susceptible), one by *S. lugdunensis*, and one by *P. bivia*. *S. aureus* 1 and 4 and *S. lugdunensis* were isolated in the SF of patients with PJIs, while *S. aureus* 2 and 3 and *P. bivia* were from native joint infections. For staphylococci, the CLSM analysis evidenced both single bacterial cells and cellular aggregates with different sizes (Figures 1, 2 and Supplementary Figure S1), ranging from less than 10 cells (*S. lugdunensis*, Figure 1d) to clumps exceeding 20 μm (*S. aureus* 4, Figure 2b). Interestingly, the largest aggregates were observed in the samples associated with prosthetic devices. Only for *S. aureus* 4, all the matrix components were efficiently stained (Figure 2). All matrix stains colocalized with SYTO 9, with a signal intensity proportional to the size of the aggregates. In the right panel of Figure 2a, WGA lectin stain is shown. However, it is not possible to discriminate whether the signal is derived from the PNAG produced by bacteria and/or from incorporated hyaluronic or from other N-acetylglucosamine (GlcNAc) containing polysaccharide. As shown in Figure 3, α-fibrin antibody could efficiently stain *S. aureus* biofilm-like aggregates, thus confirming the importance of binding to human-derived fibrin for the pathogenesis of joint infections. Copious bacterial clumps were observed in the sample infected by the coagulase-negative *S. lugdunensis*. However, those aggregates rarely outnumbered 10 cells (Figure 1d). The highest infiltration of immune cells was detected in the sample infected by *P. bivia*. Therefore, the SF was diluted in sterile saline to appreciate the presence of both prokaryotic and eukaryotic cells (Figures 4a,b). As depicted in Figure 4b, *P. bivia* was present as single cells or couples of cells, probably replicating bacteria, but no aggregate similar to those formed by staphylococci was seen throughout the whole sample.

### *In vitro* Growth in SF Induces the Formation of Macroscopic Aggregates by Staphylococci

As already shown in the recent literature, staphylococci own an incredible capability of forming free-floating aggregates visible to the naked eye in human and animal SFs in laboratory settings (Dastgheyb et al., 2015a,b,c; Perez and Patel, 2015; Ibberson et al., 2016; Gilbertie et al., 2019). To compare the characteristics of bacterial aggregates *in vitro* to those in clinical samples, the clinical isolates were cultured in sterile SFs from uninfected outpatients. *P. bivia* was grown in anaerobiosis for up to 14 days. Turbidity was visible after 8–10 days in thioglycollate broth and after 10–12 days in SF, however, no bacterial aggregates were detected in accordance with what was observed in the infected sample.

All staphylococci efficiently formed aggregates both in SF and in BSF with similar kinetics. Clumps of cells were visible to the naked eye after about 9 h of incubation for *S. aureus* strains and 12 h for *S. lugdunensis*. The formation of staphylococcal aggregates was monitored at different time points by CLSM. It was observed that, after 6 h, all staphylococcal aggregates displayed a dimension similar to that observed in the corresponding clinical sample (data not shown).

**FIGURE 5:**
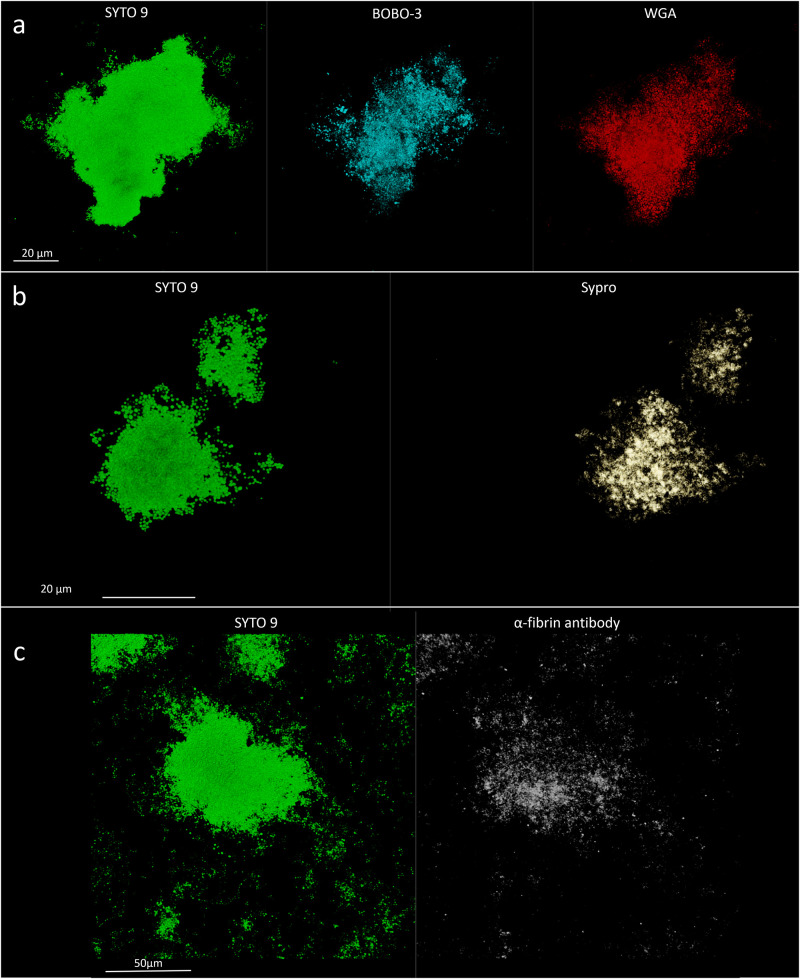
Detection of matrix components in *in vitro* assays. **(a)** *Staphylococcus aureus* 1; left panel, SYTO 9 for cellular biomass; central panel, BOBO-3 for eDNA staining; and right panel, WGA stain for GlcNAc containing polysaccharides. **(b)** *S. aureus* 1; left panel, SYTO 9 for cellular biomass; right panel, SYPRO-Ruby staining. **(c)** Detection of fibrin incorporation in *S. aureus* 2 biofilm-like aggregate; left panel, SYTO 9 for nucleic acids (green); right panel, α-fibrin antibody (gray).

**FIGURE 6:**
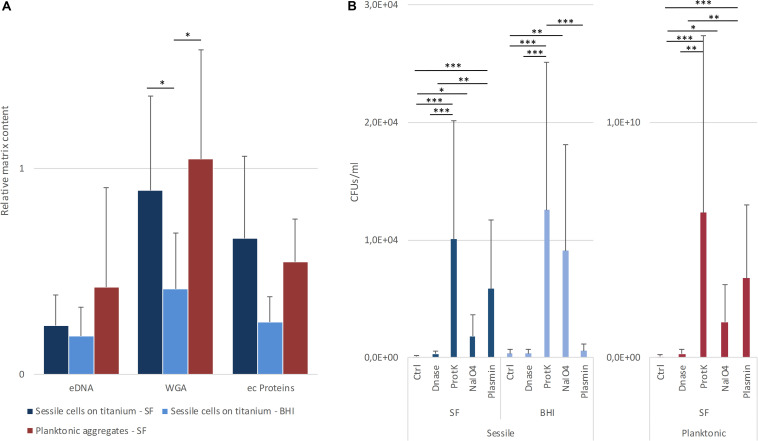
**(A)** Relative content of matrix components in sessile and planktonic cells grown in lab medium (BHI) or in synovial fluids (SF). The data reported are the average of three distinct acquisitions of three replicates for each strain. **(B)** Estimation of aggregate dispersion based on the measurement of increased colony forming units (CFU). Four replicas for each treatment were performed for each strain. *p < 0.05, **p < 0.01, and ***p < 0.001.

The matrix production was quantified in each of the acquired images (Figures 5a,b) by comparing the biomass of each stained component (WGA-stained PNAG, BOBO-3 stained eDNA, and SYPRO Ruby stained extracellular proteins) to the volume of cells stained with SYTO 9. Due to the weak aggregation of planktonic staphylococci in culture medium (i.e., BHI), the signal emitted from all the dyes was barely detectable by CLSM. Thus, data referring to bacterial aggregates cultured in BHI were not reported. A similar phenotype in terms of matrix composition was observed when comparing biofilm-like clumps and sessile biofilm grown in the presence of BHI or SF (Figure 6A). The only significant difference was in the relative content of WGA-stained polysaccharides. Indeed both planktonic aggregates and sessile biofilm displayed an increase of WGA signal (*p* < 0.01) when cultured in SF, possibly due to the incorporation of hyaluronic acid physiologically present in SF. Similarly, fibrin, which is present in SF, was incorporated in free-floating biofilm-like aggregates of all *S. aureus* and *S. lugdunensis* (Figure 5c).

### Dispersal Treatment on Sessile Biofilms and Planktonic Aggregates

The contribution of different matrix components to the stability of biofilm and bacterial aggregates was assessed by estimating the cell dispersion (increase of CFU count) after the incubation with both chemical and enzymatic treatments. As shown in Figure 6B, proteinase K was always the most efficient treatment (*p* < 0.001) in terms of cell dispersion. Interestingly, treatment with sodium metaperiodate, which hydrolyzes polysaccharides, exerted the greatest effect on the biofilms grown in BHI broth (*p* < 0.001). Even though also the sessile and planktonic aggregates grown in BSF were affected by sodium metaperiodate (*p* < 0.001), the enzymatic dispersal by plasmin provoked a major increase in CFUs in the supernatant (*p* < 0.001), confirming its important role in staphylococcal clumping. As expected, the plasmin treatment on biofilm grown in BHI was comparable to the untreated control.

### Synovial Fluid Induces a Reorganization of Biofilm Architecture on Titanium Surface

**FIGURE 7:**
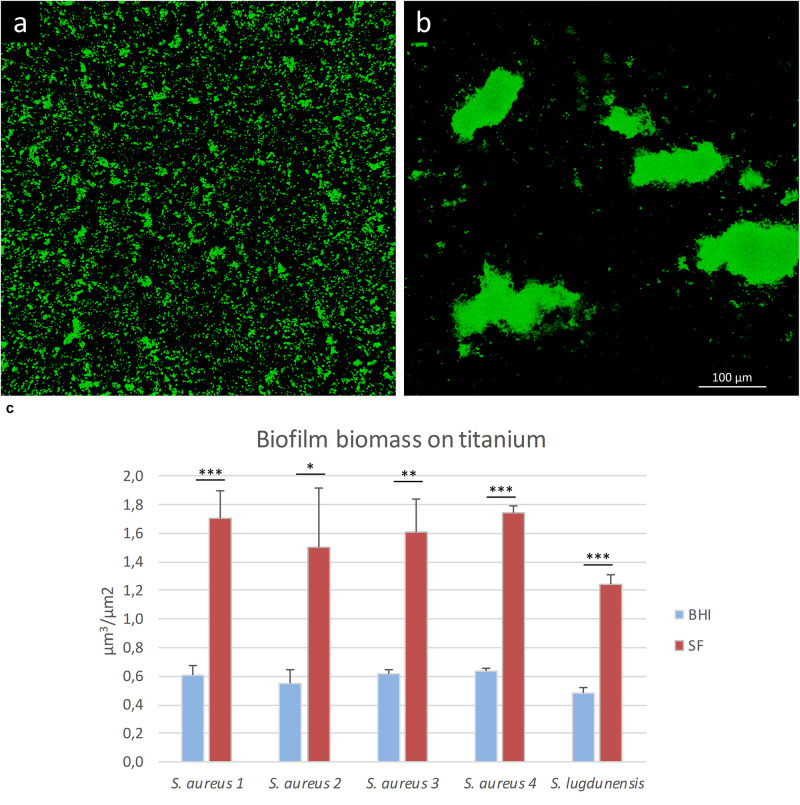
Biofilm formation on titanium alloy in the presence of lab medium or synovial fluid (SF). In this figure, *S. aureus* 1 biofilm is grown in the presence of **(a)** BHI or **(b)** SF. **(c)** Biofilm volume/surface ratio of the staphylococcal strains grown in BHI (blue bars) or SF (red bars). The data reported are the average of three distinct acquisitions for each strain. *p < 0.05, **p < 0.01, and ***p < 0.001.

To evaluate if SF can influence the organization of the biofilm architecture, staphylococcal isolates were cultured on titanium disks in the presence of either SF or BHI. As shown in Figure 5c, the matrix biomass of biofilm grown in BHI is commonly lower than that produced by bacteria grown in SF, except for eDNA. The lower matrix biomass complied with the observed three-dimensional architecture of the sessile biofilm mainly composed of small aggregates of cells that cover the whole titanium surface when grown in BHI (Figure 7a) and in big sparse clumps of cells when grown in SF (Figure 7b). Indeed the latter showed a significant increase in the ratio between the volume of biofilms and the covered titanium surface (Figure 7c), indicating a thicker biofilm biomass. Such increase varied from 2.57 (*S. lugdunensis*) to 2.8-folds (*S. aureus* 1) with respect to the counterpart grown in BHI.

### Antimicrobial Susceptibility

Before assessing the MIC and the MBC of both planktonic cells and bacterial aggregates following the CLSI guidelines, the concentration of bacteria needed to obtain aggregates with a dimension comparable to those observed in clinical SF was evaluated. After 6 h of pre-incubation, no aggregates were visible to the naked eye, but upon microscopic investigation, most of the cells had clumped to form aggregates of 5–30 μm in diameter, with the exception of *S. lugdunensis* aggregates which hardly exceeded diameters larger than 10–15 μm. The initial bacterial inocula assessed by means of resazurin assay and CFU count are reported in Supplementary Table S2.

The antibiotic susceptibility profile of the tested strains is reported in Table 1. The MIC values in Mueller Hinton (MH) broth were similar for all the strains, except for *S. aureus* 1 that resulted as resistant to oxacillin and levofloxacin. When the same assay was repeated with BSF instead of MH broth, the inhibitory concentrations rose up to four- to 32-folds in all tested strains, particularly those of rifampin and vancomycin. Furthermore, all the tested strains showed vancomycin MIC values beyond the clinical breakpoints.

**TABLE 1:** Minimum inhibitory concentration (MIC) and minimum bactericidal concentration (MBC) of oxacillin (OX), levofloxacin (LVX), rifampin (RI) and vancomycin (VA) against staphylococci in Muller Hinton (MH) broth and in bovine synovial fluid (BSF).

		MIC				MBC			
			
		OX	LVX	RI	VA	OX	LVX	RI	VA
MH	*S. aureus* 1	64	16	0.008	1	512	1,024	0.063	16
	*S. aureus* 2	0.063	0.125	0.008	0.5	1	2	0.031	8
	*S. aureus* 3	0.125	0.125	0.008	1	1	1	0.063	16
	*S. aureus* 4	0.125	0.125	0.008	1	4	2	0.125	16
	*S. lugdunensis*	0.063	0.125	0.008	2	0.25	0.25	0.063	16
BSF	*S. aureus* 1	256	256	0.25	16	< 1,024	<1,024	1	16
	*S. aureus* 2	1	1	0.25	4	16	16	1	32
	*S. aureus* 3	1	4	0.125	16	32	8	0.5	64
	*S. aureus* 4	1	2	0.25	32	64	8	0.5	64
	*S. lugdunensis*	1	0.5	0.063	32	4	4	0.125	64
BSF (pre-formed aggregates)	*S. aureus* 1	< 1,024	<1,024	2	256	< 1,024	<1,024	16	1,024
	*S. aureus* 2	16	8	1	16	256	256	4	256
	*S. aureus* 3	8	16	2	64	256	256	8	512
	*S. aureus* 4	128	128	2	64	1,024	1,024	16	1,024
	*S. lugdunensis*	1	0.5	0.125	32	32	32	2	256

*Data are expressed in mg/L.*

When *S. aureus* clinical isolates were exposed to antibiotics as pre-formed aggregates, the susceptibility to the treatment further decreased. In fact, all the tested antibiotics inhibited the growth of all *S. aureus* strains with concentrations higher than the clinical breakpoints. Contrarily, only a slight increase of MIC values was observed in the pre-formed aggregate of *S. lugdunensis*.

Also, the MBC values consistently increased, including those that completely eradicate *S. lugdunensis*.

Coherently with the inability of *P. bivia* to clump in SF, changes in its antibiotic susceptibility profile were not detected when cultured in the presence of BSF (Table 2).

**TABLE 2:** Minimum inhibitory concentration (MIC) and minimum bactericidal concentration (MBC) of vancomycin (VA), ampicillin (AM), metronidale (MZ) and imipenem (IP) against *Prevotella bivia* in thyoglycollate (Thy) and in bovine synovial fluid (BSF).

	MIC				MBC			
		
	VA	AM	MZ	IP	VA	AM	MZ	IP
Thy	4	0.125	0.5	0.063	16	0.125	0.5	0.032
BSF	4	0.125	0.5	0.032	32	0.25	0.5	0.032

*Data are expressed in mg/L.*

## Discussion

Microbes have evolved to associate together as “communities” to form complex structures encased in a polymeric matrix, known as biofilms (Costerton et al., 1999). Microbial biofilms are ubiquitous in almost every environment. These densely packed cells are able to communicate with each other to adapt to environmental changes, resist to chemical stresses and starving condition, and disperse to colonize new niches. The majority of human infectious diseases involves biofilms of bacterial pathogens, which are less susceptible to antimicrobials and to host immune system, thus providing a plausible explanation of why therapy failure is common (Hall-Stoodley et al., 2004). The introduction of a foreign body (i.e., implantable devices) is known to trigger bacterial adhesion and biofilm formation as bacteria have a preferred tropism for abiotic surfaces (Zimmerli and Sendi, 2017; Arciola et al., 2018).

Albeit the original definition of biofilm established a mandatory initial attachment to a solid surface (biotic or abiotic), recent microscopic analysis clearly demonstrated the presence of free-floating cellular aggregates in clinical samples from chronic infections (i.e., otitis media, cystic fibrosis, and chronic wounds) (Bjarnsholt et al., 2009; Homøe et al., 2009; Burmølle et al., 2010; Bay et al., 2018). These non-attached clumps of cells share many features with sessile biofilms, such as matrix components (auto-produced or environment-derived) and the ability to resist high concentrations of antimicrobials and immune cell phagocytosis. Hence, it has been proposed to include this microbial lifestyle in the biofilm definition (Alhede et al., 2011). In the last years, thorough *in vitro* researches, the incredible ability of *S. aureus* and *S. epidermidis* to form biofilm-like aggregates in SF has been reported (Dastgheyb et al., 2015a,b,c; Perez and Patel, 2015; Ibberson et al., 2016; Gilbertie et al., 2019). In 2008, in a case report describing an infected prosthetic elbow, direct imaging of periprosthetic tissue, cement spacer, and aspirated joint fluid revealed viable cells of *S. aureus* associated in biofilm aggregates (Stoodley et al., 2008). Since then, no direct evidence of free-floating clumps in SF was reported in the literature. Thus, to ascertain this microbial phenotype, joint aspirates collected from patients with suspected joint infection were investigated by means of CLSM.

In accordance with the epidemiology of joint infection (Aggarwal et al., 2014; Drago et al., 2017), staphylococci were the overwhelming majority of the infective agents encountered during this study. Compared to *S. lugdunensis*, all four *S. aureus* isolates demonstrated the capability to form larger clumps of cells. Indeed *S. aureus* is known to possess an incredible arsenal of surface clumping factors to bind host fibrinogen and fibrin, which are present in high quantities in SF. Furthermore, *S. aureus* owns at least two coagulases that promote the formation of a fibrin shield around the bacterial cells (Crosby et al., 2016). The pivotal role of fibrin incorporation in the stability of planktonic aggregates was demonstrated in various *in vitro* studies. Indeed treatment with plasmin, tissue plasminogen activator, or proteinase K was among the most efficient enzyme applications in dispersing cells (Dastgheyb et al., 2015a; Gilbertie et al., 2019). The presence of fibrin as a matrix component of biofilm-like aggregates was confirmed here by direct immuno-staining, which revealed a dense fibrin clot overlapping the cellular cluster, despite the fact that fibrin and fibrinogen were most likely present throughout the whole sample.

It is interesting to note that the largest clumps of *S. aureus* were observed in SFs aspirated from patients who had undergone a joint arthroplasty. This finding might support the notion that the introduction of foreign, abiotic surfaces might boost biofilm formation. In the case report of Stoodley and colleagues, the aggregates were first identified in the final wound debridement of a total elbow arthroplasty from a patient with a long and complicated clinical history (Stoodley et al., 2008). In that case, clumps of viable cells, with a diameter up to 100 μm, were reported, while in our sample’s aggregates did not exceed 30 μm. The dispersal of single cells or clumps of cells from a sessile biofilm is an event that has been widely investigated and represents a complex process that involves the coordination of various signals and molecular effectors (Guilhen et al., 2017). Both dispersal treatment and determination of matrix composition performed in the present study revealed almost overlapping phenotypes between free-floating aggregates and sessile biofilms grown in SF, possibly sustaining the origin of planktonic clumps from the underlying surface attached to the cells. However, the ability of staphylococci and other species to planktonically coaggregate in SF with kinetics shorter than those needed to attach to a surface matures as a biofilm and dispersion was already demonstrated (Dastgheyb et al., 2015a; Gilbertie et al., 2019). Taken together, evidence might indicate that planktonic coaggregation to form biofilm-like structures and dispersal from sessile biofilms might be two coexisting modalities of infection, in particular for those species able to scavenge host molecules.

Unfortunately, in the present study, the patient’s history and clinical data, comprising previous antibiotic treatment, are not available. This shortcoming represents a known limitation, together with the low number of infected SFs encountered in the time span of the study.

Despite the presence of implanted material and a notable bacterial load, *S. lugdunensis* displayed very small clumps of cells. Even though this species is classified as a CoNS, it is characterized by an unusual pathogenicity. Indeed *S. lugdunensis* expresses a coagulase with an activity similar to that of *S. aureus* (Becker et al., 2014) and a fibrinogen-binding protein linked to the bacterial cell wall that acts as a clumping factor (Mitchell et al., 2004). Thus, the *in vivo* phenotype of *S. lugdunensis* might be due to other host-related factors, which can be overcome by *S. aureus* strains with their incredible adaptability and virulence. In fact, *S. lugdunensis* grew *in vitro* to form very large aggregates, although not as large as those of *S. aureus* and with a slower kinetic, also demonstrating its ability to efficiently incorporate SF-derived fibrin. Even if polysaccharide intercellular adhesin locus has been identified in a number of *S. lugdunensis* isolates, biofilm formation relies predominantly on protein factors and eDNA rather than PNAG (Argemi et al., 2017). Indeed while the *S. lugdunensis* biofilm was negative for WGA staining when grown in a culture medium (Frank and Patel, 2007), in the present study, the aggregates grown *in vitro* in SF were clearly stained by WGA. This could be explained by a possible ability of *S. lugdunensis* to bind and incorporate hyaluronic acid in the matrix joint fluids. Indeed *S. lugdunensis* formed a significantly larger biofilm in the presence of hyaluronic acid than under standard laboratory conditions (Ibberson et al., 2016). In addition, hyaluronic acid was shown to be an important constituent of *S. aureus* planktonic and sessile biofilms. Hyaluronic acid could serve as hindrance to positively charged antibiotics or act as an early signal of aggregation, even if probably not contributing to clump stability as demonstrated by the hyaluronidase treatment (Gilbertie et al., 2019).

Although few other species have been shown to be able to form biofilm-like aggregates in SF (Gilbertie et al., 2019), the herein encountered *P. bivia* neither displayed tightly adhering cells in the clinical sample nor was it able to form a sessile or planktonic biofilm in the presence of both SF and culture medium. While in the past *P. bivia* was shown to bind connective tissue matrix proteins such as fibronectin and laminin (Eiring et al., 1998), other studies demonstrated a weak or almost absent biofilm-forming ability (Guaglianone et al., 2010; Patterson et al., 2010). The consequences are reflected in the lack of increase of recalcitrance to antimicrobial treatment in the presence of SF, contrarily to those species that are able to aggregate. This finding suggests that such tolerance must rely primarily on the quick grouping of neighboring cells and the presence of particular matrix components, rather than the slow growth of biofilm cells or adaptive events, as antibiotic susceptibility was found to be reversible upon disruption (Alhede et al., 2011).

In this study, two different approaches to evaluate the impact of SF on the susceptibility of antimicrobials commonly used in orthopedic practice were employed. In the first experimental setting, bacteria were inoculated concomitantly with antibiotics to mimic a possible contamination of the surgical site, in which preoperative prophylactic antimicrobial agents are usually present. Differently in the second approach, the cells were inoculated and allowed to aggregate before exposure to antibiotics to resemble the treatment of an infected native joint. Before this experiment, aggregate formation was observed with CLSM, with the obtained clumps of a size resembling those observed in the clinical samples. Here we confirmed that the growth in the presence of SF considerably affects antibiotic susceptibility, showing that bacterial cells are still able to aggregate at concentrations that would be inhibitory in standard culture conditions. In addition, the entity of recalcitrance appears to be species-specific and proportional to the size of clumps that the different species are able to form. Even though *S. lugdunensis* displayed an increase of MIC values similar to that of *S. aureus* strains in the first experimental setting, it showed a minor decrease of susceptibility when pre-aggregated cells were challenged. This is possibly due to the smaller size of aggregates that this strain was able to form in the time span before the treatment. However, the antimicrobial susceptibility of all the tested bacteria drastically decreased when assayed in SF, which represents an important issue considering that infections are likely to start from one single cell (Gerlini et al., 2014) and joint infections might arise from the contamination of sporadic cells. Furthermore, Ghimire et al. recently showed that neutrophils are unable to efficiently phagocytize staphylococcal aggregates exceeding 10 μm in diameter and that they are supposed to be recruited rapidly and in sufficient numbers to scavenge all bacterial cells and prevent them to aggregate and grow to critical dimensions (Ghimire et al., 2019).

## Conclusion

The obtained results provided evidence that staphylococci are able to form biofilm-like planktonic aggregates in both native and prosthetic infected joints, with the clumps closely resembling their sessile counterparts and able to efficiently scavenge host fibrin. While other species showed the ability to planktonically aggregate in SF, further evidences from clinical samples are needed as only three different species were identified and analyzed in the present work. The use of human- or animal-derived SF as an *ex vivo* approach for further *in vitro* investigation might improve the value of the results, bringing *in vitro* conditions closer to clinical settings. Here it has been demonstrated that the presence of SF caused a significant reorganization of biofilm architecture on titanium alloy surface, and this knowledge might have an impact on further studies for the improvement of orthopedic biomaterials. On the other hand, *in vitro* bacterial culture in SF rapidly leads to exceptionally large microbial aggregates which are several folds bigger than those observed in clinical samples and might overestimate the phenotypes observed.

## Data Availability Statement

The datasets generated for this study are available on request to the corresponding author.

## Ethics Statement

The studies involving human participants were reviewed and approved by the San Raffaele Hospital Ethical Committee (No. 146/int/2018). The patients/participants provided their written informed consent to participate in this study.

## Author Contributions

AB and ED contributed to the conception and design of the study. AB and MB collected the samples. AB and PS performed all the laboratory assays. AB wrote the first draft of the manuscript. AB, MB, PS, and ED critically revised the manuscript. All authors contributed to manuscript revision, read and approved the submitted version.

## Conflict of Interest

The authors declare that the research was conducted in the absence of any commercial or financial relationships that could be construed as a potential conflict of interest.

## Supplementary Material

FIGURE S1 Scatter plot representation of aggregate dimensions and single cells, identified in clinical samples by means of confocal laser scanning microscopy. The aggregates in each acquired image were measured with LasX software. The size reported in the graph refers to the widest distance between the cells of the same aggregate.

FIGURE S2 Gram staining of biofilm-like aggregates of *S. aureus* 1 grown in bovine synovial fluid before **(A)** and after **(B)** dispersal by combining sonication and proteinase K enzymatic treatment.
