# Supplementary figures and images for: Identification and Characterization of Planktonic Biofilm-Like Aggregates in Infected Synovial Fluids From Joint Infections
Source: Front Microbiol. 2020 Jun 30;11:1368. doi: 10.3389/fmicb.2020.01368 (PMC7344156; doi:10.3389/fmicb.2020.01368)

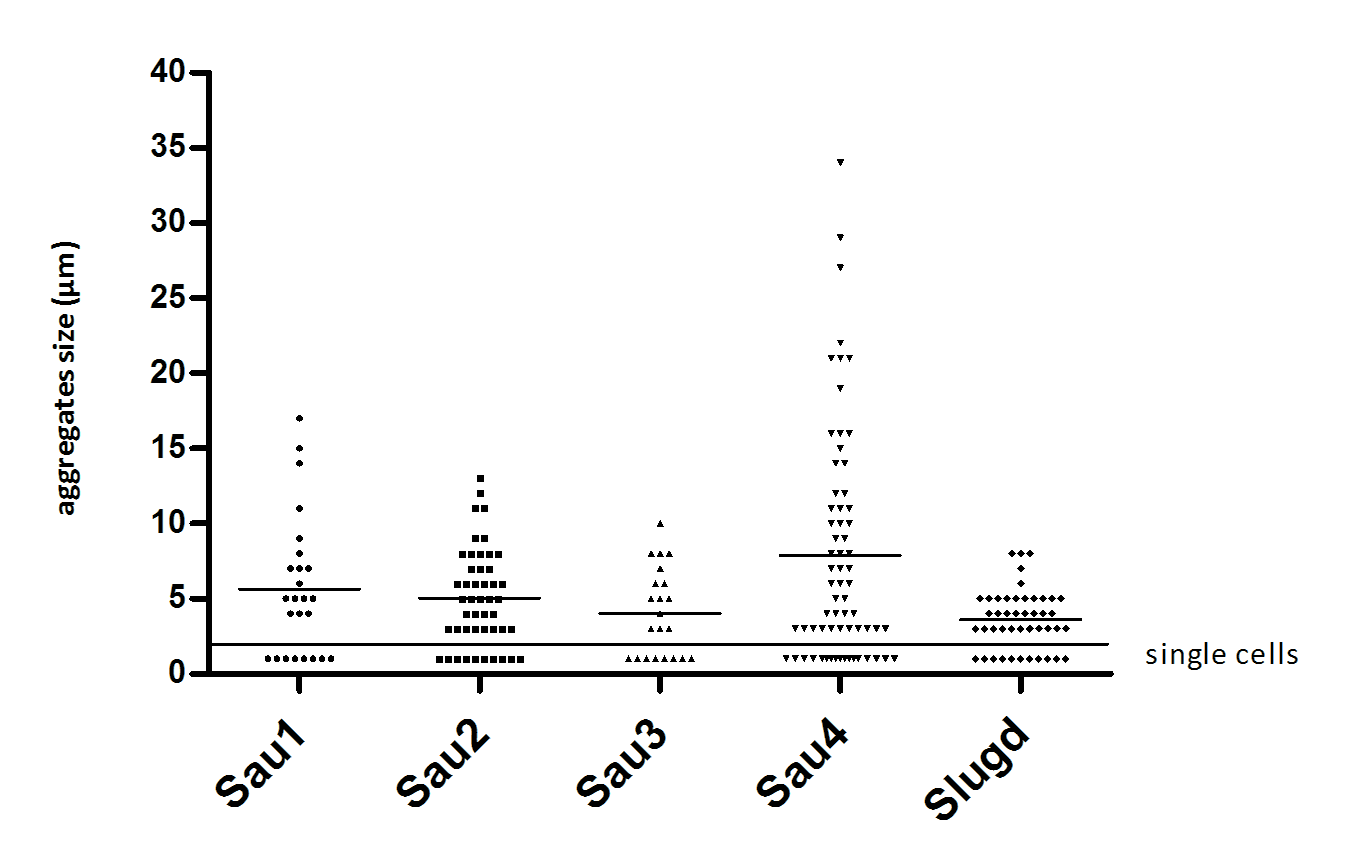

Supplement: FIGURE S1 — Scatter plot representation of aggregate dimensions and single cells, identified in clinical samples by means of confocal laser scanning microscopy. The aggregates in each acquired image were measured with LasX software. The size reported in the graph refers to the widest distance between the cells of the same aggregate. [file Image_1.TIF]

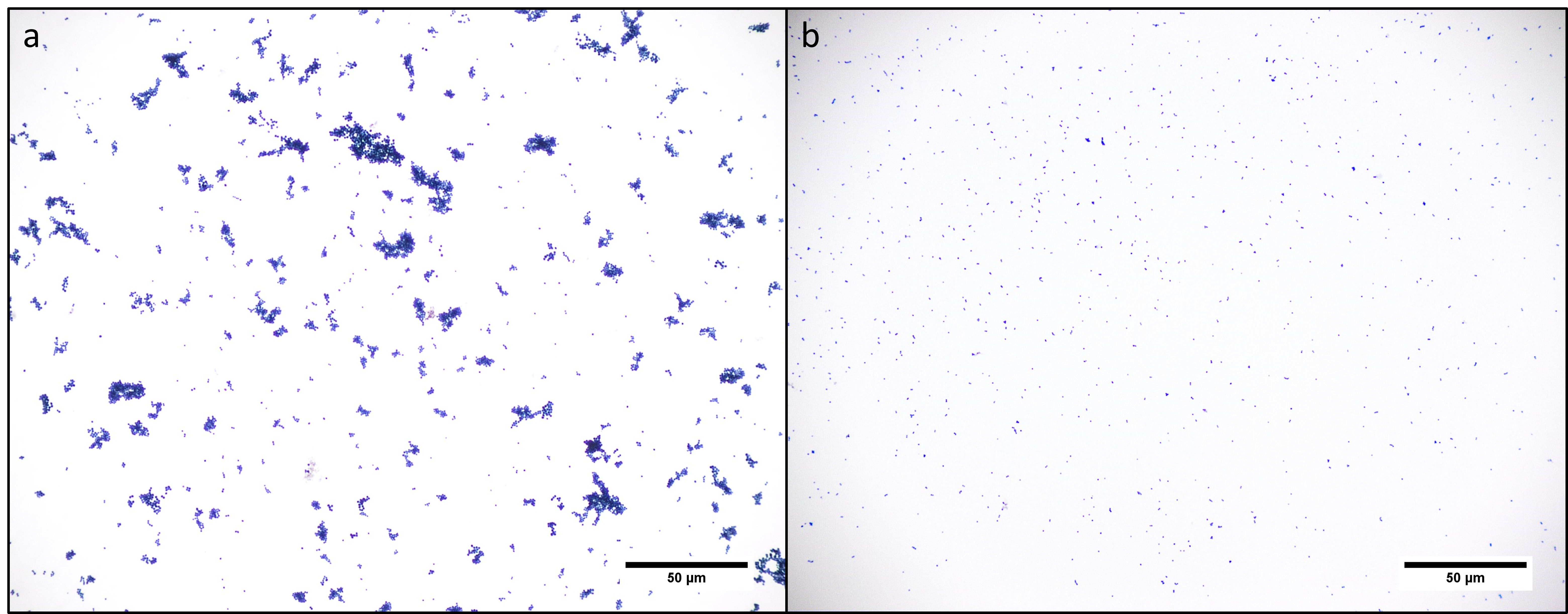

Supplement: FIGURE S2 — Gram staining of biofilm-like aggregates of S. aureus 1 grown in bovine synovial fluid before (A) and after (B) dispersal by combining sonication and proteinase K enzymatic treatment. [file Image_2.TIF]
