# Supplementary material for: Identification and Characterization of Planktonic Biofilm-Like Aggregates in Infected Synovial Fluids From Joint Infections
Source: Front Microbiol. 2020 Jun 30;11:1368. doi: 10.3389/fmicb.2020.01368 (PMC7344156; doi:10.3389/fmicb.2020.01368)
Supplement: Supplementary file 3 [file Table_1.docx]

**Table S1.** Samples information.

|  | Patient’s Age | Type of Infection | Site of Infection |
| --- | --- | --- | --- |
| *S. aureus 1* | 82 | PJI | Knee |
| *S. aureus 2* | 29 | Native Joint Infection | Knee |
| *S. aureus 3* | 88 | Native Joint Infection | Hip |
| *S. aureus 4* | 59 | PJI | Knee |
| *S. lugdunensis* | 62 | PJI | Hip |
| *P. bivia* | 34 | Native Joint Infection | Knee |
