# Supplementary material for: Identification and Characterization of Planktonic Biofilm-Like Aggregates in Infected Synovial Fluids From Joint Infections
Source: Front Microbiol. 2020 Jun 30;11:1368. doi: 10.3389/fmicb.2020.01368 (PMC7344156; doi:10.3389/fmicb.2020.01368)
Supplement: Supplementary file 4 [file Table_2.docx]

**Table S2.** Inoculum estimation for antimicrobial susceptibility assay. Cell load was measured by means of resazurin assay or CFUs count after 6h incubation in BSF. Data are expressed as CFU/ml

|  | Resazurin assay | Plate count | media |
| --- | --- | --- | --- |
| *S. aureus* 1 | 4,0E+05 | 5,8E+05 ± 1,8E05 | 4,9E+05 |
| *S. aureus* 2 | 7,5E+05 | 1,5E+06 ± 3,5E05 | 1,1E+06 |
| *S. aureus* 3 | 7,0E+05 | 1,4E+06 ± 2,9E05 | 1,0E+06 |
| *S. aureus* 4 | 8,0E+05 | 1,1E+06 ± 1,6E05 | 9,3E+05 |
| *S. lugdunensis* | 9,0E+05 | 5,6E+05 ± 6,8E04 | 7,3E+05 |
